# Nuclear Proteomics of Induced Leukemia Cell Differentiation

**DOI:** 10.3390/cells11203221

**Published:** 2022-10-14

**Authors:** Svetlana Novikova, Tatiana Tolstova, Leonid Kurbatov, Tatiana Farafonova, Olga Tikhonova, Natalia Soloveva, Alexander Rusanov, Alexander Archakov, Victor Zgoda

**Affiliations:** Laboratory of Systems Biology, Institute of Biomedical Chemistry, 119121 Moscow, Russia

**Keywords:** HL-60 cells, ATRA, induced differentiation, nuclear proteome, transcription factors, isobaric labeling, alkaline fractionation, TMT, scheduled multiple reaction monitoring, isotopically labeled peptide standards

## Abstract

Studies of induced granulocytic differentiation help to reveal molecular mechanisms of cell maturation. The nuclear proteome represents a rich source of regulatory molecules, including transcription factors (TFs). It is important to have an understanding of molecular perturbations at the early stages of the differentiation processes. By applying the proteomic quantitative profiling using isobaric labeling, we found that the contents of 214, 319, 376, 426, and 391 proteins were altered at 3, 6, 9, 12, and 72 h, respectively, compared to 0 h in the HL-60 cell nuclear fraction under all-*trans*-retinoid acid (ATRA) treatment. From 1860 identified nuclear proteins, 231 proteins were annotated as proteins with transcription factor (TF) activity. Six TFs (RREB1, SRCAP, CCDC124, TRIM24, BRD7, and BUD31) were downregulated and three TFs EWSR1, ENO1, and FUS were upregulated at early time points (3–12 h) after ATRA treatment. Bioinformatic annotation indicates involvement of the HL-60 nuclear proteome in DNA damage recognition in the RUNX1-triggered pathway, and in the p53-regulation pathway. By applying scheduled multiple reaction monitoring using stable isotopically labeled peptide standards (MRM/SIS), we found a persistent increase in the content of the following proteins: PRAM1, CEPBP, RBPJ, and HIC1 in the HL-60 cell nuclear fraction during ATRA-induced granulocytic differentiation. In the case of STAT1, CASP3, PARP1, and PRKDC proteins, a transient increase in their content was observed at early time points (3–12 h) after the ATRA treatment. Obtained data on nuclear proteome composition and dynamics during granulocytic differentiation could be beneficial for the development of new treatment approaches for leukemias with the mutated *p53* gene.

## 1. Introduction

Cell differentiation is a crucial process required for the normal functioning of multicellular organisms, especially for the maintenance of rapidly regenerating tissues, e.g., skin and mucous epithelium, fat, liver, and blood cells. The proper regulation of cell differentiation is the key process that leads to cell functionalization, and deranged maturation leads to the development of cancer, including leukemias.

The HL-60 promyelocytic leukemia cell line is related to hematopoietic precursors of granulocytes and monocytes [1]. The HL60 cell genome is characterized by amplification of the *c-myc* proto-oncogene and major deletion of the *p53* gene [2,3]. These cells could be induced to differentiate into functional neutrophils or monocytes, depending on the substance acting on them [1]. Treatment with dimethyl sulfoxide (DMSO) or all-*trans*-retinoid acid (ATRA) lead to differentiation of HL60 cells into functional neutrophils. As a metabolite of vitamin A, ATRA in physiological concentration (~2 nM) is involved in normal hematopoiesis as well [4,5]. To date, high dosage ATRA (Vesanoid^®^) (~1–10 μM) represents effective therapeutics for the treatment of acute promyelocytic leukemia (APL), the subtypes of the highly heterogeneous acute myeloid leukemia (AML). At the same time, 5-year survival rates of patients with non-APL types of AML is about 40–45% [6]. Moreover, deletion of the *p53* gene, which is associated with unfavorable prognosis, occurs in 10% of AML (non-APL types) cases [6]. The therapeutic effect of ATRA has been demonstrated for certain genetically or molecularly defined subgroups of AML (non-APL types) [5]. The understanding of the molecular mechanisms underlying granulocytic differentiation will allow identification of cell targets for potential drugs that, in combination with ATRA, could increase the effectiveness of AML (non-APL types) therapy. Thus, leukemic cell lines, namely HL-60 cells that differentiate under ATRA treatment into functional neutrophils, represent a convenient model for studies of the mechanisms implicated in cell differentiation.

A number of signaling pathways that underlie differentiation processes are triggered by various membrane or nuclear receptors. In cases of retinoic acid signaling, ATRA binds to the transport protein RBP1 and then penetrates through the cell plasma membrane and cytoplasm into the nuclei [7]. A nuclear heterodimer receptor RARα/RXR serves as a cellular target for various retinoids, including ATRA. In the absence of ligands, RARα/RXR acts as a repressor of retinoic acid response element (RARE)-containing genes. Next, ATRA binds to RARα/RXR, followed by the release of histone deacetylases (HDACs) and transcription co-repressors (N-CoR or SMRT), and by the recruitment of transcription co-activators (NcoA1/SRC-1, CBP/p300, p/CIP, and ACTR) [8]. The subsequent cascade of molecular events leads to morphological changes in myeloid precursors and their maturation into functional granulocytes. However, the whole set of effectors necessary for the implementation of such a cascade has not yet been studied in detail. At the same time, nuclear proteins are a substrate for the very first step of the ATRA-triggered differentiation program performance.

The nucleus represents the regulatory hub of the eukaryotic cell and it determines the cell fate through an intricate molecular network. In mammals, the nuclear proteome comprises at least 14% of protein-coding genes [9]. The nuclear proteome includes proteins of the nuclear matrix, transport proteins (e.g., importins and exportins), enzymes that modify chromatin to change its conformation (acetyl transferases, methyl transferases, E3 ubiquitin ligases, etc.), enzymes involved in DNA replication and transcription (e.g., RNA polymerase II), as well as proteins involved in DNA repair, and transcription factors (TFs).

TFs that activate or repress the expression of genes by interacting with genomic cis-regulatory DNA elements are a group of proteins that have attracted our particular interest. Despite the fact that in mammals, TFs are encoded by approximately 1500 genes, which makes them the second largest group of proteins after the immunoglobulin superfamily [10], qualitative and quantitative data on TF abundance are still not very exhaustive, generally due to their low expression levels. Nevertheless, the study of the protein composition of nuclei of HL-60 cells treated with ATRA would provide insight into the mechanism of regulation of the induced granulocytic differentiation.

Rapidly evolved omics technologies, e.g., whole-genome transcriptome analysis and high throughput mass-spectrometry proteomics, provide rich data on the cell molecular landscape, including organelle proteomics [11]. Notably, transcriptomic techniques are characterized by high sensitivity and allow for the detection of low-abundance molecules, such as nuclear TFs. At the same time, proteome levels reflect phenotypic features of cells more accurately. Furthermore, expression levels of transcripts and proteins, which originate from the same protein-coding gene, are poorly correlated to each other [12].

Several attempts have been undertaken to study nuclear proteins of HL-60 leukemic cells treated with pharmaceutical substances that cause differentiation or apoptosis [13,14,15]. At the transcriptome level, the time-course gene expression analyses of HL-60 cell differentiation into macrophages, under 12-O-tetradecanoylphorbol-13-acetate (TPA) treatment, revealed changes in the expression of various transcription factors, e.g., JunD, FosB, NKX2.1, and KLF4, at the early time points (0.25, 0.5, 1, 2, 3, 6, and 12 h) after TPA-treatment [13]. It has been reported that TFs JunD and FosB are important for macrophage differentiation.

At the proteome level, a study that applied 2D-PAGE coupled with Western blotting revealed the altered expressions of nuclear matrix-associated proteins PML, HSC70, and NuMA in HL-60 cells treated with etoposide [14]. The nuclear localization of apha-dystrobrevin in HL60 cells during ATRA-induced granulocytic differentiation has been determined by the 2D-PAGE /MALDI-TOF platform [15]. Finally, in our own studies, we detected proteins with TF activity, including TFs of ETS (PU.1, ERG, and ETV6) and CEBP (CEBPA, CEBPE, CEBPZ, and CEBPB) families, as well as the TF MAX, RUNX2, and RXRA in the intact HL-60 cells [16].

Many nuclear proteins, especially TFs, are low abundant, and at the same time, they perform crucial biological functions, which determine the cell fate. Therefore, enriching nuclear proteins is an important task for their efficient detection by proteomic methods. Moreover, the molecular perturbation at the early steps of a response to an inducer could involve key proteins, which may represent targets for new therapeutics.

In this study, we investigated the time-course dynamics of the HL60 nuclear proteome, with special attention to early time points after the ATRA treatment (3, 6, 9, and 12 h). To understand the molecular pathways of cell differentiation, we performed mass tag (TMT)-based proteomic profiling, combined with alkaline fractionation of digested nuclear proteins (TMT/2D), to achieve high proteome coverage and precise quantification of nuclear proteins in a time-course manner under the ATRA treatment. This resulted in the identification and quantitation of hundreds of nuclear proteins with TF activity that are involved in pathways of DNA damage recognition, RUNX1-triggered transcription, and in p53-dependent regulation. These pathways could be essential for differentiation of malignant HL60 leukemic cells into functional neutrophils.

## 2. Materials and Methods

### 2.1. Cultivation of HL-60 Cell Line

The HL-60 cells (obtained from Collection of Cell Cultures of Vertebrates ((SPBIC), Sankt-Peterburg, Russia) were cultivated in RPMI-1640 medium in the presence of 10% fetal bovine serum, 100 U/mL penicillin, 100 U/mL streptomycin and 2 mM L-glutamine (all Gibco™, Paisley, UK) in a CO2 incubator under standard conditions (37 °C, 5% CO2, 80% humidity). The 50 µM stock solution of ATRA (Sigma-Aldrich, St. Louis, MO, USA) in ethanol was prepared. Experimental HL-60 cells were subjected to the 10 µM ATRA treatment and control HL-60 cells were treated with an equal volume of the solvent (ethanol).

Applying flow cytometry, granulocytic differentiation was assessed by the CD11b and CD38 expression levels using the ZE5 Cell Analyzer (BioRad, Hercules, CA, USA). At 72 h after the ATRA treatment, the cells were collected, washed twice from medium remnants with PBS, and pelleted by centrifugation at 3000× *g* for 15 min, using an Eppendorf 5424R centrifuge (Eppendorf, Hamburg, Germany). The cell pellets were stored in liquid nitrogen until transcriptomic and proteomic analysis.

The number of cells was obtained using a cell counter and a cell viability analyzer, the TC20 Automated Cell Counter (BioRad, Hercules, CA, USA), and a cell counting kit (BioRad, Hercules, CA, USA). The HL60 cell line was checked for mycoplasma contaminations.

### 2.2. Transcriptome Analysis

Total RNA was isolated from the cells using an RNeasy Mini Kit (Qiagen, Hilden, Germany) at 0, 0.5, and 1 h after the ATRA treatment. The quality of the extracted RNA was checked using a Bioanalyzer 2100, RNA 6000 Nano LabChips, and the 2100 Expert standard software (all Agilent Technologies, Santa Clara, CA, USA). To prepare cDNA, approximately 0.5 g of each RNA sample was used in the reaction of the reverse transcription, performed using the Low RNA Input Linear Amp Kit (Agilent Technologies, Santa Clara, CA, USA) according to standard protocol. The cRNA samples for time points 0, 0.5, and 1 h after the ATRA treatment were labeled with Cy5-CTP (Perkin Elmer, Waltham, MA, USA) and the control samples (the time point 0 h) were labeled with Cy3-CTP (Perkin Elmer, Waltham, MA, USA). The cRNA fragmentations and hybridizations were performed using in situ the Hybridization Kit Plus (Agilent Technologies, Santa Clara, CA, USA) according to standard protocol. Data acquisition was performed using the DNA Microarray Scanner G2505C (Agilent Technologies, Santa Clara, CA, USA). The raw transcriptome data were processed using the Feature Extraction software (version 10.1.3.1; Agilent Technologies, Santa Clara, CA, USA).

Statistical data analysis by ANOVA with the p-value cut-off set at 0.05 was carried out using the GeneSpring GX12.5 software (Agilent Technologies, Santa Clara, CA, USA).

### 2.3. Lysis of Cells and Isolation of Nuclear Fraction

Several schedules of the HL-60 cell harvesting after the ATRA treatment have been applied in the omics time-course experiments. Most of them were focused on advanced time points after the ATRA treatment (e.g., 24, 48, and 96 h [17], 24 h and 72 h [18], 24 and 48 h [19]). In our own previous proteomic experiments, we also applied advanced time point schedules (i.e., 24, 48, and 96 h) [20,21], with the addition of only one early time point (i.e., 3 h). At advanced time points, granulocyte-specific surface markers CD11b and CD38 may be detected by cytofluorimetry that reflects the complete maturation of leukemic cells into neutrophils. In our own proteomic studies, functional annotation of differentially expressed at advanced time point (24 and 96 h) proteins also reflects myeloid cell differentiation [21]. However, the regulatory proteins that were responsible for obtaining neutrophil phenotypes already returned to the control level, and went unnoticed. To detect the proteomic onset of induced granulocytic differentiation, HL-60 cells were harvested at 0, 3, 6, 9, 12, and 72 h after the ATRA treatment. The time point of 72 h after the ATRA treatment was selected to control proper granulocyte differentiation. For proteome analysis, we performed the ATRA-induced differentiation experiments in three independent biological replicates (overall, 18 samples).

The nuclear fraction of proteins was obtained using chemical extraction [16]. To break the plasma membrane, 200 μL of ice-cold lysis buffer, containing 10 mM HEPES-NaOH (pH 7.9), 1.5 mM MgCl2, 10 mM KCl, 0.5% NP-40, 0.1 mM EDTA, 320 mM sucrose, and cOmplete™ protease inhibitors (Roche, Basel, Switzerland), was added to HL-60 cell samples, followed by the pellets’ resuspension by pipetting. Next, an additional 200 μL aliquot of the lysis buffer was added, and reaction mix was incubated for 10 min on ice. The reaction mix was carefully layered on the top of 800 μL of buffer containing 10 mM HEPES-NaOH (pH 7.9), 1.5 mM MgCl2, 10 mM KCl, 0.5% NP-40, 0.1 mM EDTA, and 1.2 mM sucrose, followed by centrifugation at 16,000× *g* for 25 min at 4 °C, using the Eppendorf 5424R centrifuge (Beckman Coulter, Indianapolis, IN, USA). The pellet was dissolved in 200 μL of buffer containing 1% SDS, 100 мM Tris-HCl (pH 8.5), and cOmplete™ protease inhibitors (Roche, Basel, Switzerland), and subjected to ultrasonication using the Bandelin Sonopuls probe (“BANDELIN electronic GmbH & Co. KG”, Berlin, Germany). The sample protein concentration was measured using a Pierce™ BCA Protein Assay Kit (Pierce, Rockford, IL, USA).

### 2.4. Sample Preparation Prior to Mass-Spectrometric Analysis

Tryptic digestion of HL60 cell proteins was performed according to the FASP protocol (filter-aided sample preparation) [22] with a slight modification. Briefly, an aliquot of each sample, containing 100 µg of total protein, was placed into concentration filters with a cut-off of 30 kDa (Merck Millipore Limited, Tullagree, Ireland) and centrifuged at 11,000× *g* for 15 min at 20 °C. For reduction and alkylation of the disulfide bonds, each sample was incubated in presence of 30 mM Tris TCEP (Thermo Fisher Scientific, Waltham, MA, USA) and 50 mM CAA (Sigma-Aldrich, St. Louis, MO, USA) at 80°C for 1 h. To remove detergent remnants, the samples were washed 3 times with a buffer that contained 8 M urea (Sigma-Aldrich, St. Louis, MO, USA) in 100 mM Tris Cl, at pH 8.5. Prior to tryptic digestion, samples were washed twice with 50 mM TEAB, at pH 8.5, then subjected to centrifugation at 11,000× *g* for 15 min at 20 °C. Next, 50µL of 50 mM TEAB buffer (Sigma-Aldrich, St. Louis, MO, USA), pH 8.5, and trypsin (Promega, Fitchburg, WI, USA) at an “enzyme to total protein” ratio of 1:70 was added to each sample. The samples were incubated with trypsin overnight at 37 °C. The peptides were obtained by centrifugation at 11,000× *g* for 15 min at 20 °C, and the filter was washed twice with 50 µL of 5% formic acid to quench tryptic digestion. The peptide concentration was measured by the colorimetric method using a Pierce Quantitative Colorimetric Peptide Assay kit (Pierce, Rockford, IL, USA), according to standard protocol. The peptides were dried and dissolved in 0.1% formic acid to a final concentration of 3 µg/µL. To check sample loading prior to TMT-labeling, the peptide samples were subjected to high resolution mass spectrometric analysis.

### 2.5. TMT Labeling

For TMT labeling, 15 μg peptides of each sample (pre-dried in a vacuum concentrator) were reconstituted in 100 mM TEAB (pH 8.5) to a concentration of 0.2 μg/μL. TMT-10 reagent (Thermo Scientific, Waltham, MA, USA, cat# 90406) was resuspended in anhydrous ACN to a concentration of 19.5 μg/μL. The appropriate TMT reagent was added to each sample at the 1:35 reagent/peptide (wt/wt) ratio, and incubated for 1 h at room temperature. The labeling design is shown in Table S1 and in Figure S1 (“TMT labeling design”). To quench the reaction, 5 % hydroxylamine was added and samples were incubated for 15 minutes at room temperature. Labeled samples that corresponded to the time points (0, 3, 6, 9, 12, and 72 h) were combined within each biological replicate, resulting in 3 samples for MS/MS analysis. The three pooled samples were dried via vacuum centrifugation, and desalted using a C18 Stage-Tip method. The peptides were dissolved in 0.1% formic acid to a final concentration of 3 µg/µL.

### 2.6. Reversed Phase Fractionation in Alkaline Conditions

The peptides’ fractionation was performed on an Agilent 1200 Series HPLC system, equipped with a degasser, dual micro flow pump, autosampler, UV detector, fraction collector, and column compartment. The aliquot of each sample, containing 100 µg of peptides, was separated on the C18- XBridge, Waters analytical column (4.6 × 250 mm, 5 µm pore size, Waters, Ireland). The samples were loaded at a flow rate of 0.75 ml/min for 3 min in an isocratic mode of Mobile Phase A (15 mM ammonia acetate in HPLC grade water, pH 9.0). Then, the peptide fractions were eluted with a gradient of Mobile Phase B (80% acetonitrile, 15 mM ammonia acetate in HPLC grade water, pH 9.0) at a flow rate of 0.75 mL/min. Fraction collection started at the 3rd minute and ended at the 23rd minute. One fraction volume was 1.5 ml; the fraction collector operated in the time slices mode. It started to collect a new fraction every 2 minutes. Overall, 10 fractions were collected in 1 duty cycle.

### 2.7. Shotgun Mass Spectrometry

The peptide samples were analyzed using the Ultimate HPLC system (Thermo Scientific, Waltham, MA, USA) connected to the Q Exactive HF-X Quadrupole-Orbitrap, equipped with a nanoelectrospray ion source (Thermo Scientific, Waltham, MA, USA). Peptide separations were performed on a RP-HPLC Zorbax 300SBC18 analytical column (C18 3.5 µm, 75 µm inner diameter and 150 mm length, Agilent Technologies, Santa Clara, CA, USA), using a linear gradient from 98% Mobile Phase A (water, 0.1% formic acid) and 2% Mobile Phase B (water, 0.1% formic acid, and 80% acetonitrile) to 30% Mobile Phase B over 78 min, at a flow rate of 0.4 µL/min. High resolution mass spectrum acquisition was carried out in the positive ion mode using the Orbitrap analyzer. The resolution was set at 60,000 (m/z = 400) and at 15,000 (m/z = 400) for the MS and MS/MS scans, respectively. The AGC target of 10^6^ and 10^5^ with a maximum ion injection time of 50 ms and 100 ms was used for MS and MS/MS spectrum acquisition, respectively. Up to 20 of the most abundant precursors were used for triggering MS/MS spectra. To obtain fragment ions, the higher energy collisional dissociation (HCD) was applied, and normalized collision energy of 30 units was used. The signal threshold for MS precursor ions was set to 50,000 for an isolation window of 2 m/z width. To increase proteomic coverage, dynamic exclusion of fragmented precursors was used with a repeat count 1, repeat duration of 10 s, and exclusion duration of 60 s. Singly charged precursors and precursors with undefined charge states were excluded from triggering the MS/MS scans. Dionex™ Cytochrome C Digest (Thermo Scientific, Waltham, MA, USA) was used for quality control LC-MS/MS runs.

### 2.8. Protein Identification, TMT-Based Quantitation, Data Annotation and Bioinformatics Analysis

For identification and TMT-based quantification, mass spectrometry data were processed in the MaxQuant software (version 2.0.3.0, Max Planck Institute of Biochemistry, Martinsried, Germany) [23]. Mass spectrometric data were interpreted using the built-in Andromeda algorithm. Identification was performed using the FASTA file (UP000005640, UniProt Release 2022_02, 20,598 proteins, EMBL-EBI, Hinxton Cambridge, UK) and its inverted counterpart to calculate the false discovery rate (FDR) level. The built-in database of potential contaminants was used alongside the target FASTA file. The carbamidomethylation of cysteine was used as a fixed modification, and methionine oxidation and N-terminal acetylation were set as variable modifications. Trypsin was applied as protease, and two missed cleavages were allowed. The max mass error of 20 ppm for the precursor and fragment ions was allowed. For proteins and peptides, the FDR threshold value was set at 0.01. Quantitative analysis was performed on the basis of the reporter ion (MS2) intensity value using the MaxQuant (version 2.0.3.0, Max Planck Institute of Biochemistry, Martinsried, Germany) built-in algorithm. All working channels were assigned as references and used for weighted median normalization. Matching between runs (MBR) function was applied.

Summed signals for all proteins in each TMT channel were calculated, and the TMT channel with the highest summed signal was determined. Normalization factors that represent the ratio of highest summed signals to summed signals of each TMT channel were calculated. Sample loadings were corrected through multiplication of reporter ion intensity for each protein by TMT channel-specific normalization factors.

Potential contaminants, false positive identifications, and proteins identified only by peptides that contained variable modifications were removed from the proteomic dataset prior to quantitative analysis. The statistical analysis was carried out using the Perseus 1.6.0.7 software (Max Planck Institute of Biochemistry, Martinsried, Germany). Protein groups with more than 30% missing values in total across all channels were omitted from further analysis. Two-sample t-tests were used to assess differences between the two groups. The data were corrected for multiple comparisons using permutation-based FDR with a threshold value of 0.001, S0 = 0.1. Only the proteins identified by at least 2 unique peptides were used for quantification.

Venn diagrams were generated using the Python3 script using the venn3 library. The STRING database v.11.0 (https://cn.string-db.org/) (accessed on 15 September 2022) [24] was utilized to find the protein–protein interactions (PPIs). A high (0.7) and the highest (0.9) confidence score were used. The experiments, data on gene fusion and curated databases were used as active interaction sources. The built-in functional enrichment analysis was performed. Functional annotation results according to the Gene Ontology (GO) and Reactome Pathways databases were used for visualization. For functional annotation, the bioinformatic tools DAVID v.6.8 (DAVID Bioinformatics Resources, MD, USA) [25] and PANTHER v.17.0 (https://panther.com/) (accessed on 15 September 2022) [26] were used. The volcano plot was constructed using the VolcaNoseR web app (https://huygens.science.uva.nl/VolcaNoseR/) (accessed on 15 September 2022) [27]. The box plots were generated using BoxPlotR, a web tool for the generation of box plots (http://shiny.chemgrid.org/boxplotr/) (accessed on 15 September 2022) [28].

### 2.9. Multiple Reaction Monitoring (MRM)

The standard peptides were synthesized using the solid-phase technique on the Overture™Robotic Peptide Library Synthesizer (Protein Technologies, Manchester, UK), according to the published method [29]. The isotopically labeled lysine (13C6,15N2), arginine (13C6,15N4) or leucine (13C6,15N1) were used for isotopically labeled peptide synthesis, instead of the unlabeled counterparts (Table S1, “SIS Peptides”). Concentrations of the standard peptides were determined using the acidic hydrolysis of peptides, followed by amino acid analysis with fluorescent signal detection, as described previously [30].

The aliquot of each sample that contained 5 µg of total peptide was injected for the LC/MRM run. The LC/MRM experiment was performed in three technical replicates for each sample. Prior to LC/MRM analysis, the samples were dried in a vacuum concentrator and dissolved in 0.1% formic acid spiked with SIS, in an equimolar concentration of 500 fmol/µL. The final content of each SIS was 50 fmol/µg of the total peptides. The peptide samples were separated using an Agilent 1200 series HPLC system (Agilent Technologies, Santa Clara, CA, USA), coupled with a TSQ Quantiva triple quadrupole mass analyzer (Thermo Scientific, Waltham, MA, USA). The peptides were loaded on the ZORBAX SB-C18 analytical column (150 × 0.5 mm, 5 µm particle diameter) (Agilent Technologies, Santa Clara, CA, USA) and eluted in a gradient of acetonitrile, with a flow rate of 20 µL/min. First, the column was equilibrated with 5% Mobile Phase B (80% acetonitrile in 0.1% formic acid) and 95% Mobile Phase A (0.1% formic acid) for 5 min. Next, the concentration of Mobile Phase B was linearly increased to 50% for 30 min. To remove peptide remnants, Mobile Phase B was increased to 99% in 1 min, and the column was washed with 99% Mobile Phase B for 5 min. To equilibrate the column, the concentration of Mobile Phase B was returned to the initial conditions for 1 min, and the column was balanced for 9 min. Mass spectrometry analysis was carried out in the scheduled multiple reaction monitoring (MRM) mode, using the following settings: the capillary voltage was set at 4000 V, the velocity of the drying gas (nitrogen) was 7 L/min, the velocity of the axillary gas (nitrogen) was 5 L/min and the capillary temperature was 300 °C. The isolation window of 0.7 Da was used for the first and third quadrupole. The scan cycle time was 1.2 s with a dwell time of 40 ms per transition, and the collision gas (argon) pressure in the second quadrupole was set at 1.5 mTorr. The retention time window was a 3 min width for each precursor ion. The transitions and normalized collision energy values (V) are listed in Supplementary Table S1 (“MRM Table”).

The results were processed and plotted using SkylineMacCoss Lab Software (version 4.1.0, University of Washington, Seattle, WA, USA). The peak area ratio for the endogenous peptide and the corresponding SIS standard was automatically compared in Skyline. To obtain the amount of protein, the ratio calculated in Skyline was multiplied by the known content of each SIS standard. The content of each protein was taken as the mean value of the MRM/SIS measurements obtained in three technical replicates. The result was expressed in fmo of target protein per µg of total protein.

## 3. Results

### 3.1. Flow Cytofluorometry Showed CD38 and CD11b Upregulation under the ATRA Treatment

**Figure 1:**
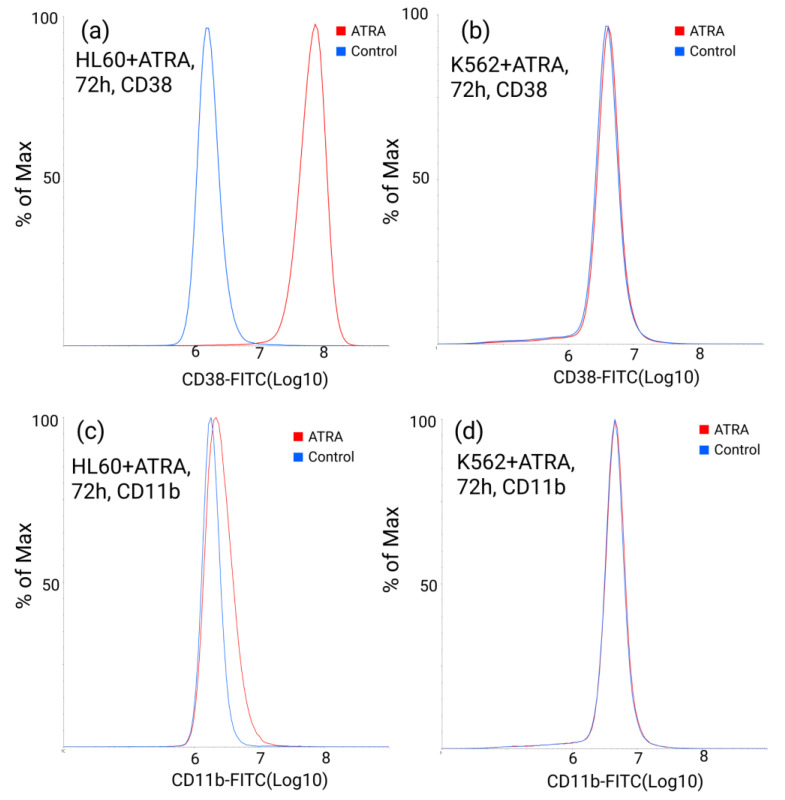
Flow cytometric assessment of the granulocytic marker CD38 surface expression (**a**) in all-*trans*-retinoid acid (ATRA)-treated HL-60 cells (red), compared to non-treated HL-60 cells (blue), and (**b**) in ATRA-treated K562 cells (red), compared to non-treated K562 cells (blue) at 72 h after the ATRA treatment. Flow cytometric assessment of granulocytic marker CD11b surface expression (**c**) in ATRA-treated HL-60 cells (red), compared to non-treated HL-60 cells (blue), and (**d**) in ATRA-treated K562 cells (red), compared to non-treated K562 cells (blue) at 72 h after the ATRA treatment.

To validate HL-60 cell maturation into neutrophils prior to transcriptome and proteome analysis, the expression of surface granulocytic markers CD11b and CD38 was assessed at 72 h after the ATRA treatment. We applied flow cytofluorometry using antibodies against CD11b and CD38. Expression levels of CD11b and CD38 were also measured in the control K562 cell line, which is insensitive to the ATRA treatment (Figure 1).

The mean fluorescence from HL-60 cells at 72 h after the ATRA-treatment was 2 orders of magnitude higher for the CD38 protein. A moderate increase in CD11b expression at 72 h after the ATRA treatment compared to the untreated control was also detected. At the same time, CD38 and CD11b protein levels remained unaltered in the ATRA-insensitive K562 cells. The results of flow cytofluorometry demonstrated that granulocyte differentiation of the HL-60 cells was successful.

### 3.2. Transcriptomic Profiling of HL-60 Cells at 0.5 and 1 Hour after the ATRA Treatment Indicated Modulation of Transcription Factors and the IL-10 Pathway

**Figure 2:**
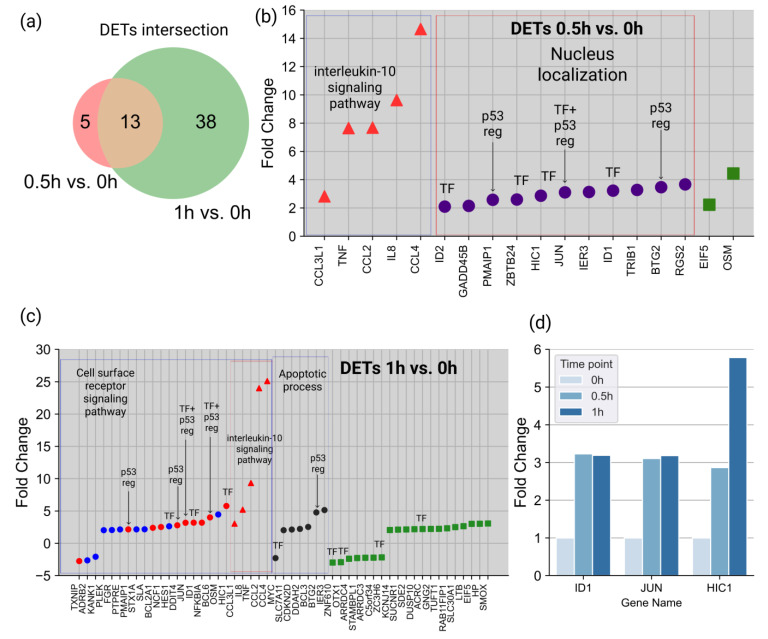
Transcriptomic profiling of whole-cell lysate of HL-60 cells at 0.5 and 1 h after ATRA treatment. (**a**) Venn diagram shows intersection of differentially expressed transcripts (DETs) at 0.5 and 1 h after the ATRA treatment. DETs expression differences (fold change > 2, *p*-value < 0.05) (**b**) at 0.5h and (**c**) at 1 h after the ATRA treatment compared to control. Red triangles indicate DETs involved in the interleukin-10 signaling pathway, purple circles indicate DETs with nuclear localization, blue circles indicate DETs involved in the cell surface receptor signaling pathway (GO:0007166), black circles indicate DETs involved in the apoptotic process (GO:0006915) and red circles indicate DETs involved in both the cell surface receptor signaling pathway and the apoptotic process. TF indicates DETs with transcription factor activity (PC00264), p53 reg with an arrow indicates DETs that are involved in p53 regulation (R-HSA-3700989). (**d**) TFs ID1, JUN, and HIC1 expression differences (fold change > 2, *p*-value < 0.05) at 0, 0.5, and 1 h after ATRA treatment.

To reveal immediate molecular perturbation, we performed whole-genome transcriptomic profiling of HL-60 cells at 0.5 and 1 h after the ATRA treatment. Overall, 18 and 51 differentially expressed transcripts (DETs) (fold change (FC) > 2, *p*-value < 0.05) were revealed at 0.5 and 1 h, respectively, after the ATRA treatment (Supplemental Table S2). These included thirteen DETs (BTG2, CCL2, CCL3L1, CCL4, EIF5, HIC1, ID1, IER3, IL8, JUN, OSM, PMAIP1, and TNF), which overlapped between the two time points studied in the transcriptomic experiment (Figure 2a).

At 0.5 h after the ATRA treatment, 5 out of 18 DETs (CCL2, CCL4, CCL3L1, IL8, and TNF) were assigned to the interleukin-10 signaling pathway (according to the Reactome pathway category, DAVID annotation) (Figure 2b). In addition, 11 out of 18 DETs (BTG2, GADD45B, HIC1, ID1, ID2, IER3, JUN, PMAIP1, RGS2, TRIB1, and ZBTB24) were assigned to nuclear localization (according to the GO database Cell Component category (DAVID annotation)). Moreover, at 0.5 h after the ATRA treatment, five DETs (ID1, ID2, HIC1, JUN, and ZBTB24) were annotated as gene-specific transcriptional regulators (PC00264), according to the Protein Class category Panther annotation (Figure 2b).

At 1 h after the ATRA treatment, there was a group of five DETs, which were assigned to the interleukin-10 signaling pathway (CCL2, CCL4, CCL3L1, IL8, and TNF) (Figure 2c). Additionally, DETs were assigned to the apoptotic process (GO:0006915) and cell surface receptor signaling pathway (GO:0007166) groups. We did not find any significantly enriched DETs localized in the nuclei at 1 h after the ATRA treatment. However, 10 DETs (ACRC, BCL6, HES1, HIC1, ID1, JUN, MYC, OTX1, ZC3H6, and ZNF610) were annotated as gene-specific transcriptional regulators (PC00264), according to the Protein Class category Panther annotation. Three TFs (ID1, JUN, and HIC1) were upregulated at two time points (Figure 2d), and in the case of HIC1, the increase in the expression levels at 1 h was almost six-fold higher compared to 0 h after the ATRA treatment.

### 3.3. Functional Annotation of HL-60 Nuclear Proteome Revealed Proteins with Transcription Regulator Activity

Prior to the time-course proteomic investigation of ATRA-induced HL-60 cell differentiation, we verified the performance of the nuclear isolation procedure by label-free proteomic profiling of leukemic cell whole lysates (WHL) and the nuclear fraction (NF). Among 1499 proteins (identified by at least two unique peptides, FDR < 0.01), 635 proteins were significantly enriched in the nuclear fraction compared to WHL (Supplementary Figure S2, Supplementary Table S3). Among them, 539 proteins (88.6%) demonstrated nuclear localization according to the GO database Cell Compartment (CC) category (Supplementary Table S3).

In order to reveal differences in nuclear protein abundances at the early steps of induced granulocytic differentiation, we performed TMT-labeling coupled with alkaline fractionation (TMT/2D), using the HL-60 cell nuclear fractions obtained at 0, 3, 6, 9, 12, and 72 h after the ATRA treatment. We identified 1860 proteins with high confidence (at least two peptides per protein, FDR < 0.01) (Supplementary Table S4). Among them, 1310 proteins (74.1%) demonstrated nuclear localization according to the GO database CC category.

To find the biological meaning of all the identified proteins, we performed functional annotation according to molecular function, regulatory pathways, and protein–protein interactions using the DAVID v.6.8, PANTHER v.17.0, and STRING database v.11.0 software.

**Figure 3:**
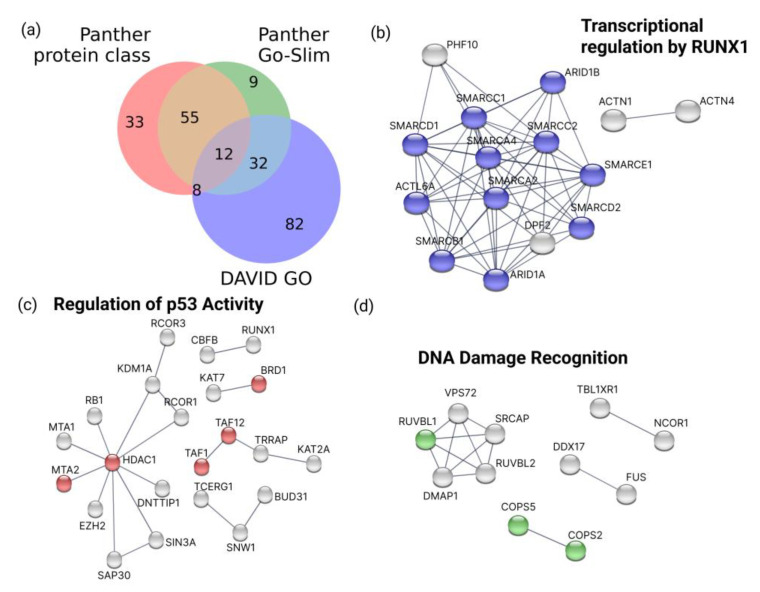
Results of bioinformatic analysis of proteins identified in the HL-60 nuclear fraction by MS/MS mass spectrometry. (**a**) Venn diagram shows proteins recognized as gene-specific transcriptional regulators (PC00264) according to the Panther protein class (Panther), as proteins with transcription regulator activity (GO:0140110) according to the Panther Go-Slim Molecular Function (Panther Go-Slim), and as proteins with transcription factor activity and protein binding (GO:0000988) according to the GeneOntology (GO) Molecular Function (DAVID). Results of analysis by STRING database v.11.0 (the highest confidence score (0.9), experiments and fusion genes as active interaction sources; k-means clustering into three clusters) are also shown. (**b**) Cluster 1 (input of 86 TFs) was enriched with interactions (PPI enrichment p-value: < 10^-16^), and with proteins involved in transcriptional regulation by RUNX1 (blue, according to the Reactome pathway). (**c**) Cluster 2 (input of 72 TFs) was enriched with interactions (PPI enrichment *p*-value: 3.11 × 10^-13^), and with proteins involved in the regulation of TP53(p53) activity (red, according to the Reactome pathway). (**d**) Cluster 3 (input of 73 TFs) was enriched with interactions (PPI enrichment p-value: 9.76 × 10^-14^ ), and with proteins involved in DNA damage recognition in GG-NER (green, according to the Reactome pathway.

Thus, 1860 proteins identified in the 2D/TMT experiment were assigned to the Molecular Function (MF) category of the GO database by the DAVID v.6.8 tool. As a result, 134 proteins were attributed to proteins with transcription factor (TF group) activity (GO:0000988~transcription factor activity, protein binding) (Figure 3a).

Moreover, 108 out of the identified 1860 were assigned to proteins with transcription regulator activity (GO:0140110), according to the Panther Go-Slim Molecular Function (Panther v.17.0) (Figure 3a). Another 108 proteins were annotated as gene-specific transcriptional regulators (PC00264), according to the Panther protein class (Figure 3a). Overall, 231 out of 1860 proteins (12.4%) involved in the regulation of transcription were identified in the HL-60 nuclear fraction in the course of ATRA induced cell differentiation (Supplementary Table S4). Among them, we found well-known regulatory families of TFs, such as CEBPs (CEBPA, CEBPB, CEBPE, and CEBPZ), RUNXs (RUNX1, RUNX2, and RUNX3), FOXKs (FOXK1 and FOXK2), STAT1, SMAD4, JUNB, and MAX.

An interaction analysis of 1860 nuclear proteins by STRING showed 3 main clusters. The identified nuclear proteins were involved in transcriptional regulation by RUNX1, the regulation of TP53 (p53) activity, and DNA damage recognition in the first (Figure 3b), second (Figure 3c), and third (Figure 3c) clusters, respectively.

### 3.4. Molecular Dynamics of Proteins with Transcription Regulator Activity in HL-60 Cell Nuclear Fraction under Atra Treatment

Based on the quantitative 2D/TMT proteomic analysis, 214, 319, 376, 426, and 391 proteins were recognized as differentially expressed nuclear proteins (DENPs) at 3, 6, 9, 12, and 72 h, respectively, after the ATRA treatment (Supplementary Table S4).

Only two transcript–protein pairs overlapped between the 0.5–1 h DET and 3–72 h DENP datasets (Supplementary Figure S3). Eukaryotic translation initiation factor 5A-1 (EIF5), which was upregulated at the transcriptome levels at the earliest time points studied (0.5 and 1 h), was also observed with increased expression at the proteome level at 9 and 12 h after ATRA treatment. Moreover, neutrophil cytosolic factor 1 (NCF1) was upregulated at 1 h and at 72 h at the transcriptome and proteome levels, respectively.

**Figure 4:**
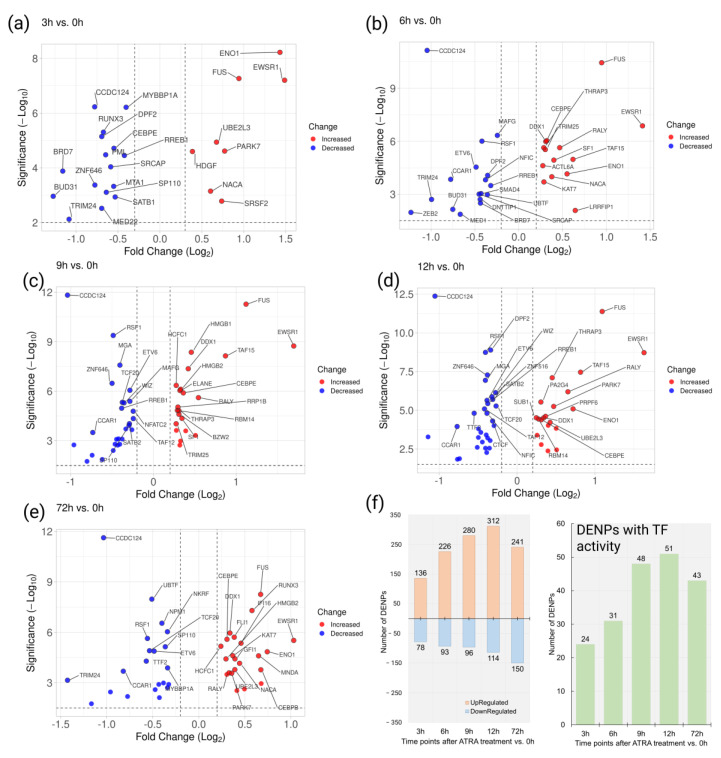
Results of proteomic quantitative 2D/TMT-profiling of the HL-60 nuclear fraction during ATRA-induced granulocytic differentiation. (**a–e**) Scatter plots show the differences in abundance for nuclear proteins with transcription factor (TF) activity at 3, 6, 9, 12, and 72 h after the ATRA treatment, respectively. Significantly up- and downregulated proteins (FDR < 0.005, S0 = 0.1) are shown by red and blue dots, respectively. In the cases of time points 9, 12, and 72 h, names are shown for the top thirty most differentially expressed nuclear proteins (DENPs). (**f**) Number of all DEPs and DEPs with TF activity at 3, 6, 9, 12, and 72 h after the ATRA treatment.

Moreover, 24, 31, 48, 51, and 43 DENPs were annotated as proteins with TF activity (Figure 4).

Overall, 42 TFs were downregulated at the early time points studied (3, 6, 9, and 12h) compared to the control. According to STRING, the downregulated TFs are involved in the regulation of TP53 (p53) activity through acetylation (HSA-6804758) (TFs BRD7, BRD1, and PML), and in BCL2L11 (BIM) transcription, regulated by RUNX3 (HSA-8952158) (TFs RUNX3 and SMAD4) (Figure S4).

Furthermore, thirty-three TFs were upregulated at least at one early time point studied (3, 6, 9, and 12h) compared to the control. Among the upregulated TFs, eight proteins were involved in DNA repair (GO:0006281) (ACTL6A, DDX1, HMGB1, HMGB2, KAT7, NUCKS1, PARK7, and TRIM25). The results of STRING analysis demonstrated the enrichment of interaction (Figure S5).

It was observed that six TFs (RREB1, SRCAP, CCDC124, TRIM24, BRD7, and BUD31) were downregulated and three TFs (EWSR1, ENO1, and FUS) were upregulated at all early time points studied (3, 6, 9, and 12h). We designated them as the core TFs with reduced and increased regulation.

### 3.5. Bioinformatic Analysis Indicates p53-Associated Regulation of HL-60 Cell Differentiation, despite p53 Gene Deletion

The HL-60 cell genome contains some characteristic mutations, i.e., extensive deletion of the *p53* gene, which leads to a lack of expression of the corresponding oncosuppressor protein [2]. Functional annotations of the proteins identified by LC-MS/MS in the HL-60 nuclear fraction revealed a group of proteins related to p53 functioning. For example, 21 proteins (BLM, BRD4, BRD7, CDKN2AIP, CHD8, EEF2, EHMT2, HDAC1, HSPD1, KDM1A, PSME3, RNF20, SETMAR, SMARCA4, SMARCB1, TAF1, TAF9, TP53BP1, TP53RK, TRIM24, and USP7) were recognized as p53 binding proteins (GO:0002039:), according to the GO database.

The identified nuclear proteins with TF activity (MTA1, HDAC1, TAF1, TAF12, BRD7, BRD1, and PML) were involved in the regulation of TP53 (p53) activity by acetylation (HSA-6804758), according to the Reactome Pathway database (Figure 3c and Supplementary Figure S4). The content of three of the above-mentioned proteins (BRD7, BRD1, and PML) decreased in response to the ATRA treatment in our experiments.

Finally, we used manual curation of proteins relevant to the p53 pathway and its main regulator MDM2 protein based on published reviews [31,32] (Table 1).

As a result, regulatory proteins of the p53 pathway involved in the DNA damage response (DDB2 and XPC), apoptosis regulation (BAX), and cell cycle progression (CCNB1) were identified in the LC-MS/MS experiments. Moreover, the proteins that regulate p53/MDM2 proteins at the transcription (TFs FLI1 and SMAD4) and post-translational modification (SSB, ATM, CSNK1D, HAUSP, and GNL3) levels were detected in the HL-60 nuclear fraction. Furthermore, the expression levels of ATM, GNL3, FLI, and SMAD4 were altered under the ATRA treatment.

### 3.6. Targeted Quantitative Mass-Spectrometric Analysis of Regulatory Proteins in the Nuclear Fraction of HL-60 Cells under the ATRA Treatment

**Figure 5:**
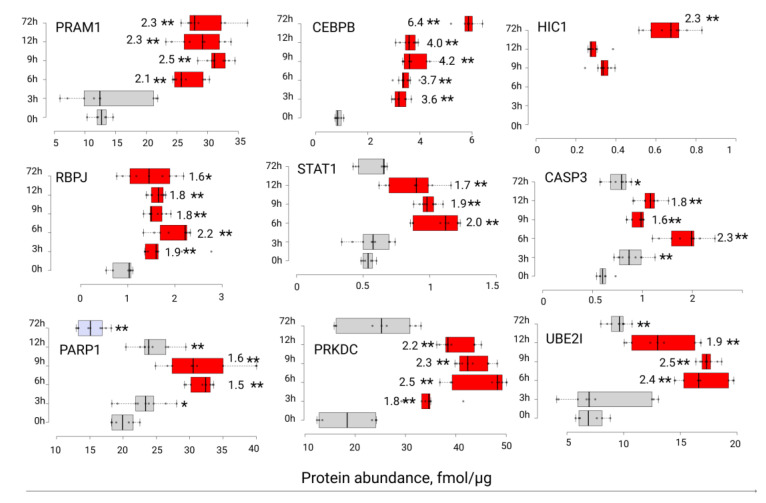
Results of quantitative MRM/SIS analysis of proteins PRAM1, CEBPB, HIC1, RBPJ, STAT1, CASP3, PARP1, PRKDC, and UBC9 in the HL-60 cell nuclear fraction at 0, 3, 6, 9, 12, and 72 h after ATRA treatment. Upregulated, unchanged and downregulated proteins are shown in red, gray, and blue, respectively. One (*) and two asterisks (**) indicate the *p*-value thresholds of 0.05 and 0.01, respectively. The number of nearby asterisks indicates the protein abundance fold change compared to the control (0 h), except for HIC1 (fold change in protein abundance compared to first time point of detection (9 h)). The x-axis shows the protein abundance in fmol/µg. The experimental time points (0, 3, 6, 9, 12, and 72 h) are shown on the y-axis. The proteins were considered as upregulated if the fold change was above 1.5.

We exploited a highly sensitive and specific MRM/SIS method for the quantification of several putative regulatory proteins (CASP3, CEBPB, HIC1, PARP1, PRAM1, PRKDC, RBPJ, STAT1, and UBE2I) in the HL-60 cell nuclear fraction at 0, 3, 6, 9, 12, and 72 h after ATRA treatment (Figure 5). Based on TMT/2D mass-spectrometric profiling, PRAM1 and CEBPB were upregulated at 72 h after the ATRA treatment, while the protein UBE2I was upregulated at 12 h after the ATRA treatment. The transcription factor HIC1 was one of the immediately altered molecules under the ATRA treatment, according to the transcriptomic data. Moreover, the proteins CASP3, HIC1, PARP1, PRKDC, RBPJ, STAT1, and UBE2I were the components of the model transcriptome- and proteome-based regulatory networks, which were obtained in our previous study of ATRA-induced HL-60 differentiation [21].

Protein levels of the PML-RARA-regulated adapter molecule 1(PRAM1) were significantly upregulated at 6 h (2.1-fold increase, *p*-value < 0.01) after the ATRA treatment, and upregulation was sustained up to 72 h (2.3-fold increase, *p*-value < 0.01). A similar expression pattern was observed for the transcription factor CCAAT/enhancer-binding protein beta (CEBPB), with significant upregulation of protein abundance (3.6-fold increase, *p*-value < 0.01), starting at 3 h and continuing up to 72 h (6.4-fold increase) after the ATRA treatment. The levels of the transcription regulator recombining binding protein suppressor of hairless (RBPJ) were also significantly upregulated (1.9-fold increase, *p*-value < 0.01), starting at 3 h and continuing up to 72 h (1.7-fold increase, *p*-value < 0.05). The protein levels of the transcription factor hypermethylated in cancer 1 protein (HIC1) were below the detection limits up to 9 h after the ATRA treatment. Then, HIC1 expression levels increased 2.3-fold (*p*-value < 0.01) from 0.3 ± 0.03 fmol/µg of total protein (9 h) up to 0.7 ± 0.08 fmol/µg of total protein (72 h).

The results of the quantitative measurements of proteins STAT1, CASP3, PARP1, PRKDC, and UBE2I revealed a peculiar arc-wise expression pattern. For transcription factor STAT1, its maximum abundance was observed at 6–12 h (two-fold increase, *p*-value < 0.01) after the ATRA treatment, followed by a return to the control time point levels at 72 h. The same pattern was observed for Caspase-3 (CASP3) (1.6–2.3-fold increase at 6–12 h) and for the SUMO-conjugating enzyme UBC9 (UBE2I) (1.9–2.5-fold increase at 6–12 h). The levels of DNA-dependent protein kinase catalytic subunit (PRKDC) were upregulated at 3–12 h after the ATRA treatment (1.8–2.5-fold increase). Finally, the content of Poly (ADP-ribose) polymerase 1 increased 1.5-fold at 6 and 9 h after the ATRA treatment. There was also a trend toward decreased abundance at 72 h after the ATRA treatment (*p*-value < 0.01, fold change was below 1.5 threshold). At the same time, the difference in protein PARP1 abundance at 72 h was two-fold lower compared to the 6–9 h time point.

## 4. Discussion

For decades, the HL-60 cell line was considered as a convenient model for studying granulocytic differentiation under the ATRA treatment. Despite high sensitivity to the ATRA treatment, HL-60 cells have wild-type *RARa*, while the *p53* gene is extensively deleted, and the *MYC* oncogene is amplified [2,3]. This suggests the existence of alternative molecular mechanisms that lead HL-60 cells to acquire granulocytic phenotypes under the ATRA treatment. Keeping in mind that the major molecular events involved cell nuclei, we performed proteomic profiling of the HL-60 nuclear fraction under the ATRA treatment in a time-course manner, paying special attention to the early time points (3, 6, 9, and 12 h after the ATRA treatment).

This work is a continuation of our previous studies on induced granulocytic differentiation of HL60 cells [20,21]. Applying the optimized protocol of nucleus isolation, alkaline peptide fractionation, together with protein quantitation by TMT, we confidently identified almost a thousand proteins that were not detected in our previous studies (Figure S6). Moreover, the bioinformatics annotation showed that most of them localized in nuclei and functionally belonged to the “binding nucleic acids” category (Figure S7). Thus, the results of current proteomic profiling reflect the dynamics of hundreds of proteins with potential regulatory functions during ATRA-induced differentiation of the HL60 cell line.

Previously, we modeled transcriptome- and proteome-based regulatory networks in silico by using the molecular composition of HL60 whole lysates as input data [21]. The expanded picture of the proteomic landscape that was obtained in the present study (806 additional proteins) experimentally confirmed the expression at the protein level of fourteen proteins involved in granulocytic differentiation, including regulatory TFs (ILF3, MEF2D, NFATC1, NFATC2, CUX1 (CDP), MAX, RBPJ, RUNX2, STAT1, and YY1), and intermediated molecules (KAT2A (GCN5), UBE2I) predicted in silico.

The TMT/2D proteomic profiling revealed numerous DENPs in HL-60 cells under the ATRA treatment. The functional annotation of proteins with altered abundance under ATRA treatment gave unexpected results. The proteomic signature associated with p53 regulation was revealed. The core downregulated TFs BRD7, RREB1, and TRIM24 were involved in p53 pathway regulation [33,34,35] and in double-strand break repair that resulted from genotoxic stress (SRCAP), which is related to p53 functioning [36]. The p53-knockdown resulted in a decreased abundance of BRD7 [34]. Experiments on p53- and BRD7-knockdown cells also revealed target genes shared by p53 and BRD7, including an important cell cycle regulator p21 (CDKN1A) [34]. *P53* gene silencing led to decreased levels of TRIM24 alongside p53 targets, e.g., MDM2 and p21 (CDKN1A) [35]. It was also shown that RREB-1 could affect apoptosis in p53-null cells [33].

Furthermore, transcripts upregulated at the earliest time points after ATRA treatment (ID1, HIC1, and JUN) were also functionally related to the p53 pathway. The HIC1 protein is involved in p53 regulation through its deactivation of SIRT1 deacetylase, which is responsible for p53 degradation. A recent study demonstrated that HIC1 can promote the repair of DNA double-strand breaks by interaction with MTA1 in a p53-independent manner [37]. Notably, the MTA1 protein was found to be downregulated in the present study. TF JUN is believed to play a role in tumor promotion via the activation of cell cycle progression or inhibition of apoptosis induced by p53 or a FAS ligand [38]. However, the controversial role of JUN in the regulation of p53 and its structural and functional homologues (e.g., p73) has been observed. It was shown that JUN enhanced p73 expression and protected it from proteasome-mediated degradation, which led to increased sensitivity to chemotherapy [39]. Early upregulation of TF ID1 under ATRA treatment promotes cell cycle progression through the inactivation of cyclin-dependent kinase inhibitor 1A (p21). In such a way, ID1 also impairs the p53-mediated response to DNA damage [40].

As mentioned above, the HL-60 cells harbor a major deletion of the *p53* gene that makes them p53-null equivalents. The absence of p53 mRNA expression in the HL-60 cells was confirmed in our previous experiments using whole-genome transcriptome analysis [21]. The curious observation of the “ghost” p53 pathway suggests the molecular bypass that uses the same effector molecules.

The main functions of the p53 pathway consist of maintaining DNA damage control and inducing cell cycle arrest and apoptosis. Each of these functions can be controlled separately through various molecular mechanisms. In the case of DNA damage control, the functional wild-type p53 protein is stabilized in response to replication stress produced by deregulated oncogenes. In addition to the oncosuppressor *p53* gene deletion, the HL60 cells harbor protooncogene *MYC* amplification, which can lead to MYC high expression levels and replication stress. [3]. It could be assumed that in the HL-60 cells, the fight against replication stress is carried out by an alternative regulator instead of the oncosuppressor p53, but uses the same molecular effector as the p53-triggered pathway.

The expression changes in the nuclear proteins related to the p53 pathway could indicate that other molecular effectors became more important. Candidates for such a role may be p53 structural analogs, e.g., the p73 protein, or alternative signaling pathways, for which the biological endpoints are DNA repair and apoptosis.

Interestingly, the HL60 nuclear proteins with TF activity were enriched by members of the RUNX1 pathway that interplayed with the p53 pathway [41,42]. In the present study, all three members of the RUNX family (RUNX1, RUNX2, and RUNX3) were identified in the HL60 nuclear fraction by mass spectrometry, and RUNX3 regulating cell cycle entry was shown to be altered under the ATRA treatment. Moreover, recent studies have demonstrated the role of RUNX proteins in the transcription-independent DNA damage response through the FANCI/FANCD2 complex [42]; both members were identified in the HL60 nuclear proteome in this study. Notably, double-knockout of RUNX1, and RUNX3 led to a DNA repair defect and leukemia predisposition in the p53-proficient mouse cells [43]. The RUNX1 and RUNX3 upregulation was observed in 53-null mouse bone-marrow cells [44]. Considering that RUNX3 loss leads to decreased transcription of the p53-dependent target gene, RUNX3 activation may provoke the response of p53 target genes. The other curious observation is the downregulation in the ATM kinase level in the ATRA-induced HL60 nuclear proteome. It was shown in the mouse model that in p53-deficient cells, suppression of ATM sensitizes tumors to DNA-damage-based chemotherapy [45]. At the same time, ATM acts as a sensor for oncogene-induced replication stress (which may be caused by MYC amplification) and transmits signals to RUNX3 for cell cycle control [41].

The TMT labeling approach is a precise and sensitive method for relative protein quantification. However, as with other isobaric labeling techniques, TMT labeling is affected by the so-called “ratio compression” issue [46]. This effect is caused by frequent precursor ion co-isolation and co-fragmentation during MS/MS analysis, leading to the detection of the signals from altered proteins against the background of unaltered ones. As a result, the underestimation of quantitative fold change ratios can be as high as 3.5-fold [47]. This observation is consistent with the results of MRM/SIS analysis compared to TMT/2D proteomic profiling for nuclear proteins CEBPB (TMT/2D FC 1.5 vs. MRM FC 6.4 at 72 h after the ATRA treatment), UBE2I (TMT/2D FC 1.3 vs. MRM FC 1.9 at 12 h after the ATRA treatment), and PRAM1 (TMT/2D FC 1.4 vs. MRM FC 2.2 at 72 h after the ATRA treatment). The “ratio compression” should be taken into consideration when assessing the extent of perturbation of DENPs with TF activity during ATRA-induced granulocytic differentiation.

The ATRA is regarded as effective therapeutics for the treatment of APL, one of the subtypes of AML, with complete remission rates of 90% [48]. Much of this success is connected to the targeted nature of the ATRA action, as it directly interacts with the chimeric receptor PLM-RARα/RXR. However, chemotherapeutic treatment with other types of AML achieves 5-year survival rates in only about 40–45% of cases [6]. At the same time, approximately 10% of AML (non-APL) cases are characterized by the deletion of the *p53* gene that is associated with adverse prognosis [6]. The HL-60 cells that also carry the deletion of the *p53* gene serve as a good model for research. Core downregulated TFs RREB1 and TRIM24, which were associated with p53 functioning, were studied in the context of leukemia. It was shown that RAS-responsive element-binding protein 1 (RREB1) was overexpressed in AML patient blasts and myeloid leukemia NB4 and HL-60 cells. Moreover, its knockdown promotes granulocytic differentiation [49]. Similarly, TRIM24 knockdown leads to antitumor effects in AML cells, and a low expression level of TRIM24 is considered a marker of favorable prognosis [50]. At the same time, downregulated TF BRD7 that transcriptionally regulates p53 pathway components is less studied in the context of leukemia. Nevertheless, upregulation of BRD7 was observed in blood cells derived from patients with acute leukemia [51].

Core downregulated proteins TRIM24, BRD7, SRCAP, and RREB1 are also involved in the regulation of nuclear androgen receptors (AR) as activators (TRIM24, BRD7, BUD31, and SRCAP) [52,53,54] or repressors (RREB1) [55]. In hormone-dependent prostate and breast cancers, AR activation leads to tumor progression. A recent study has shown that in AML patients, AR overexpression is associated with better survival [56], but the role of AR in AML biology remains unclear. In terms of the nuclear receptors, TRIM24 serves as a corepressor of RARα, which is the molecular target of ATRA. Moreover, by possessing protein-ubiquitin ligase activity, TRIM24 is involved in the proteasome-mediated degradation of the above-mentioned nuclear receptor RARα [57]. TRIM24 pharmacological targeting could provide an antileukemic effect. Notably, TRIM24 bromodomain inhibitors were developed as putative therapeutics for solid tumors [58].

Therefore, the proteins identified in the nuclear fraction of HL-60 cells that also harbor the deletion of the *p53* gene may be potential targets for the development of alternative methods of AML (non-APL types) therapy.

It should be noted that the nuclei isolation based on centrifugation could be prone to contamination with untargeted organelles, e.g., mitochondria. Partially, we managed to solve this problem by using a sucrose cushion in the process of nuclei isolation. However, for more effective purification of nuclear proteins, affinity isolation methods can be used, e.g., DNA affinity chromatography.

The ATRA, as a vitamin A derivative, is also involved in normal myeloid cell differentiation, especially at developmental stages in vertebrates [59]. Significant dysfunction of neutrophils was observed in healthy mice on a vitamin A-deficient diet [60]. In adult mammals, retinoids play a major role in the resolution of inflammation and senescence of the normal immune system [61,62]. There is a lack of data on the proteome response of normal myeloid precursors to ATRA treatment. Future studies on nuclear protein perturbation under retinoid treatment in healthy cells could shed light on the fundamental mechanism of immune system functioning.

## 5. Conclusions

Response to external stimuli, as well as the implementation of the genetic program that determines cell function in a multicellular organism, begins in the nucleus. Under ATRA treatment, nuclear proteins play a pivotal regulatory role. Applying transcriptomic and TMT/2D proteomic profiling, we investigated the dynamics of the HL-60 cell nuclear proteome under ATRA treatment in a time-course manner. Studying early time points (3–12 h) after ATRA treatment proved to be very useful in unveiling the molecular onset of induced granulocytic differentiation. We revealed differentially expressed nuclear proteins that possess transcription factor activity. The components of the nuclear proteome were shown to be involved in p53 pathway regulation, RUNX1 signaling, and DNA damage repair. Using the SIS/MRM technique, we quantified TFs HIC1, CEBPB, RBPJ, and STAT1 in the HL-60 nuclear fraction with high precision. The results indicate that proteins, which are associated with p53 signaling, despite extensive deletion of the *p53* gene, play a major role in ATRA-induced differentiation of HL-60 cells. However, the precise architecture of the putative molecular mechanism needs to be revealed. The TFs HIC1, TRIM24 and RUNXs are thought to be the most promising targets for validation of their role in the induced granulocytic differentiation of leukemic cells in vivo. The obtained nuclear proteome could be expanded by further improvement of nuclear protein isolation, e.g., by applying DNA affinity chromatography.

## Figures and Tables

**Table 1:** Proteins involved in the regulation of p53 and MDM2 protein.+—detected in proteomic experiment, ↑—increased expression, ↓—decreased expression.

Protein Name	Uniprot AN	Gene Name	Regulatory Role	Proteomics
DNA damage-binding protein 2	Q92466	DDB2	DNA damage response, p53 regulation	+
DNA repair protein complementing XP-C cells	Q01831	XPC	DNA damage response, p53 regulation	+
Apoptosis regulator BAX	Q07812	BAX	Apoptosis, p53 regulation	+
G2/mitotic-specific cyclin-B1	P14635	CCNB1	Cell cycle progression, p53 regulation	+
Friend leukemia integration 1 transcription factor	Q01543	FLI1	MDM2 transcription regulation	+(↑72 h)
Mothers against decapentaplegic homolog 4	Q13485	SMAD4	MDM2 transcription regulation	+(↓6, ↓12, and ↓72 h)
Lupus La protein	P05455	SSB	MDM2 regulation	+
Serine-protein kinase ATM	Q13315	ATM	p53 phosphorylation	+(↓3, ↓6 h)
Casein kinase I isoform delta	P48730	CSNK1D	p53 phosphorylation	+
Ubiquitin carboxyl-terminal hydrolase 7	Q93009	HAUSP	p53 and MDM2 deubiquitination	+
Guanine nucleotide-binding protein-like 3	Q9BVP2	GNL3	Prevents MDM2 ubiquitination	+(↓72 h)

## Data Availability

High-resolution mass-spectrometric data are available via ProteomeXchange with identifier PXD035281. The MRM/SIS mass spectrometric data have been uploaded to the PASSEL repository (dataset PASS02773). Transcriptomic data have been uploaded to the ArrayExpress repository (dataset E-MTAB-12026).

## Supplementary Materials

The following supporting information can be downloaded at: https://www.mdpi.com/article/10.3390/cells11203221/s1. Figure S1: Proteomic experiment design; Figure S2: The heatmap visualization of the results of label-free proteomic profiling of leukemic cell whole lysate (WHL) and nuclear fraction (NF); Figure S3: The Venn diagram shows intersection of differentially expressed transcripts (DETs) and differentially expressed nuclear proteins (DENPs) during ATRA-induced differentiation of HL-60 cell line; Figure S4: The results of the interaction analysis of 42 downregulated TFs by STRING; Figure S5: The results of the interaction analysis of 33 upregulated TFs by STRING; Figure S6: Contribution of mass-spectrometric datasets to the coverage of the proteome of HL60 cell line under ATRA treatment [20,21]; Figure S7: The 1D-heatmap visualization of the functional annotation of 806 nuclear proteins that were uniquely identified in the present study, compared to previously investigated HL60 proteomes; Table S1: TMT-labeling design, MRM transition table and list of SIS peptides; Table S2: Differentially expressed transcripts (DETs) with fold-change equal to or above 2 (*p*-value < 0.05) at 0.5 h and 1 h after ATRA treatment; Table S3: The results of label-free proteomic profiling of leukemic cell whole lysate (WHL) and nuclear fraction (NF). Functional annotation of proteins upregulated in leukemic cell NF compared to WHL, according to the GO database Cell Compartment (CC) category; Table S4: Summary of proteomic profiling of HL-60 cells under ATRA treatment. List of all nuclear proteins identified with high confidence (at least 2 peptides per protein, FDR < 0.01) (“All_proteins”). Functional annotation of nuclear proteins identified with high confidence, according to the GO database Cell Compartment (CC) category (“DAVID_GO_CC”). Lists of nuclear proteins with TF activity according to the GO database Molecular Function, Panther Go-Slim Molecular Function, and Panther protein class categories (“Proteins with TF activity”). Lists of differentially expressed nuclear proteins (DENPs) in HL-60 cells at 3, 6, 9, 12, and 72 h after ATRA treatment compared to control.
