# Supplementary material for: Nuclear Proteomics of Induced Leukemia Cell Differentiation
Source: Cells. 2022 Oct 14;11(20):3221. doi: 10.3390/cells11203221 (PMC9600443; doi:10.3390/cells11203221)
Supplement: Supplementary file 1 [file cells-11-03221-s001.zip › Supplementary materials.pdf]

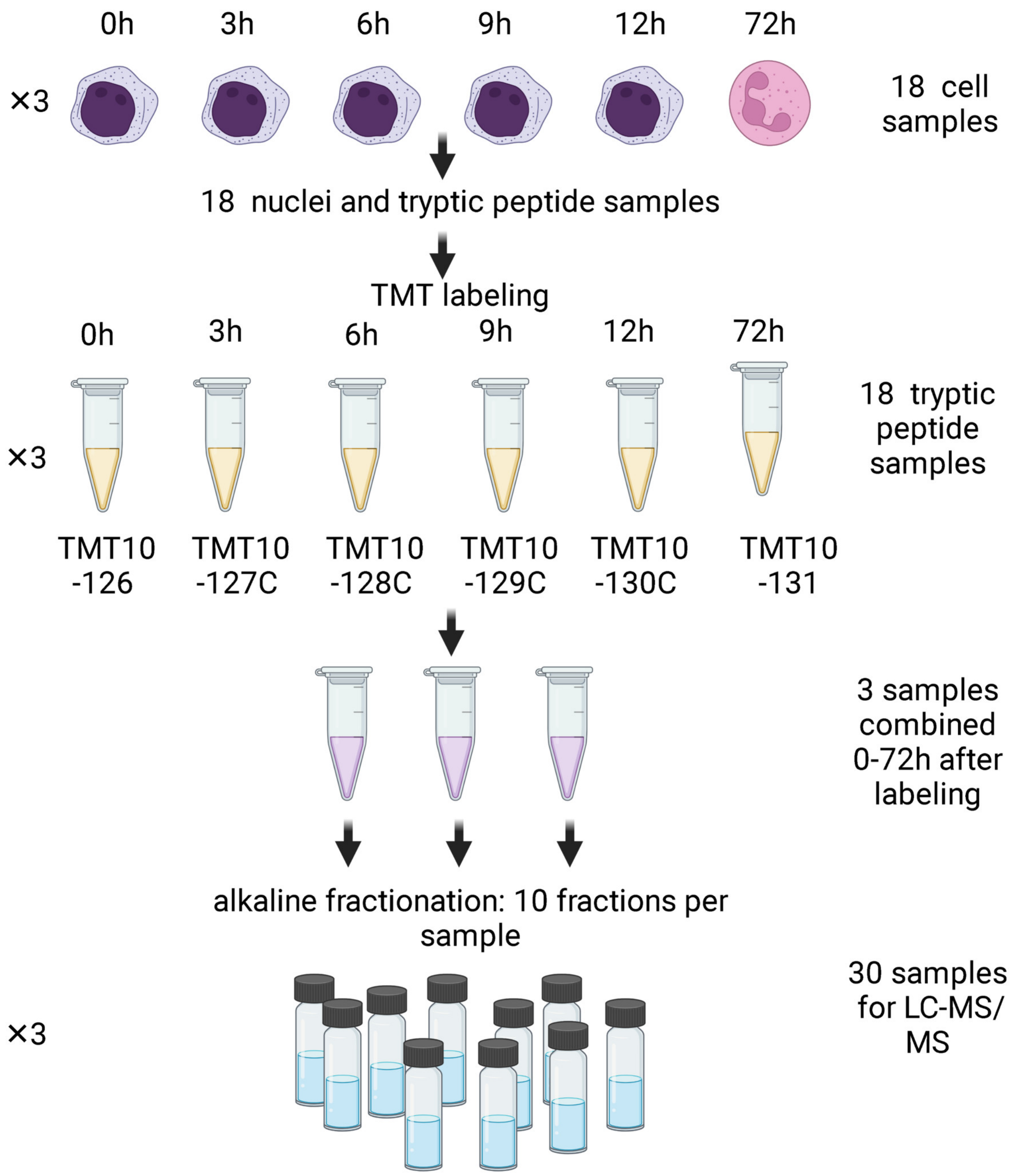

Figure S1. Proteomic experiment design.

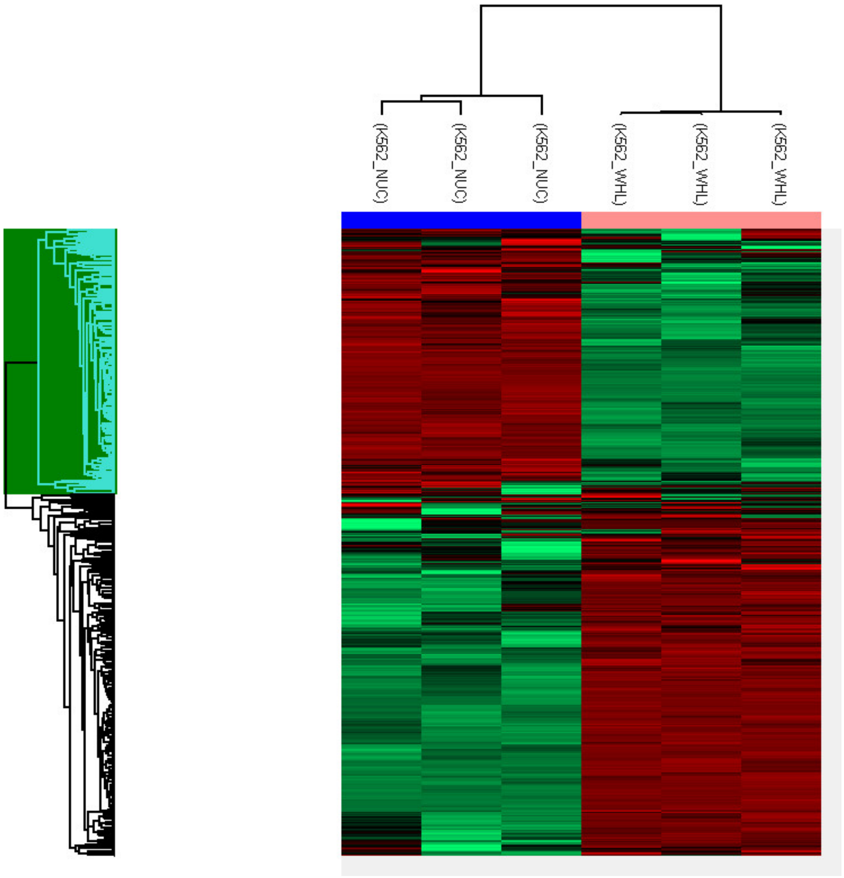

Figure S2. The heatmap visualization of the results of label-free proteomic profiling of leukemic cell whole lysate (WHL) and nuclear fraction (NF).There were two distinctive clusters of proteins with increased abundance in NUC (highlighted in the figure) or WHL samples of leukemic cells.

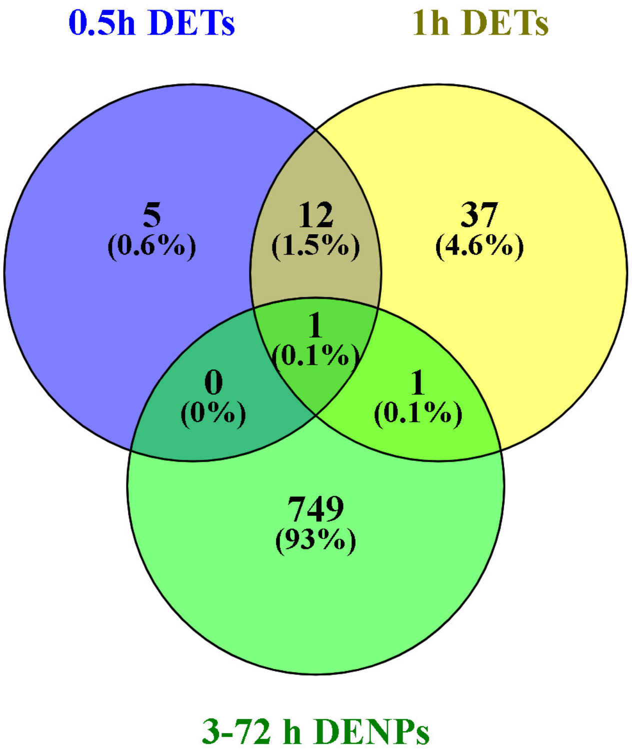

Figure S3. The Venn diagram shows intersection of differentially expressed transcripts (DETs) and differentially expressed nuclear proteins (DENPs) during ATRA-induced differentiation of HL60 cell line.

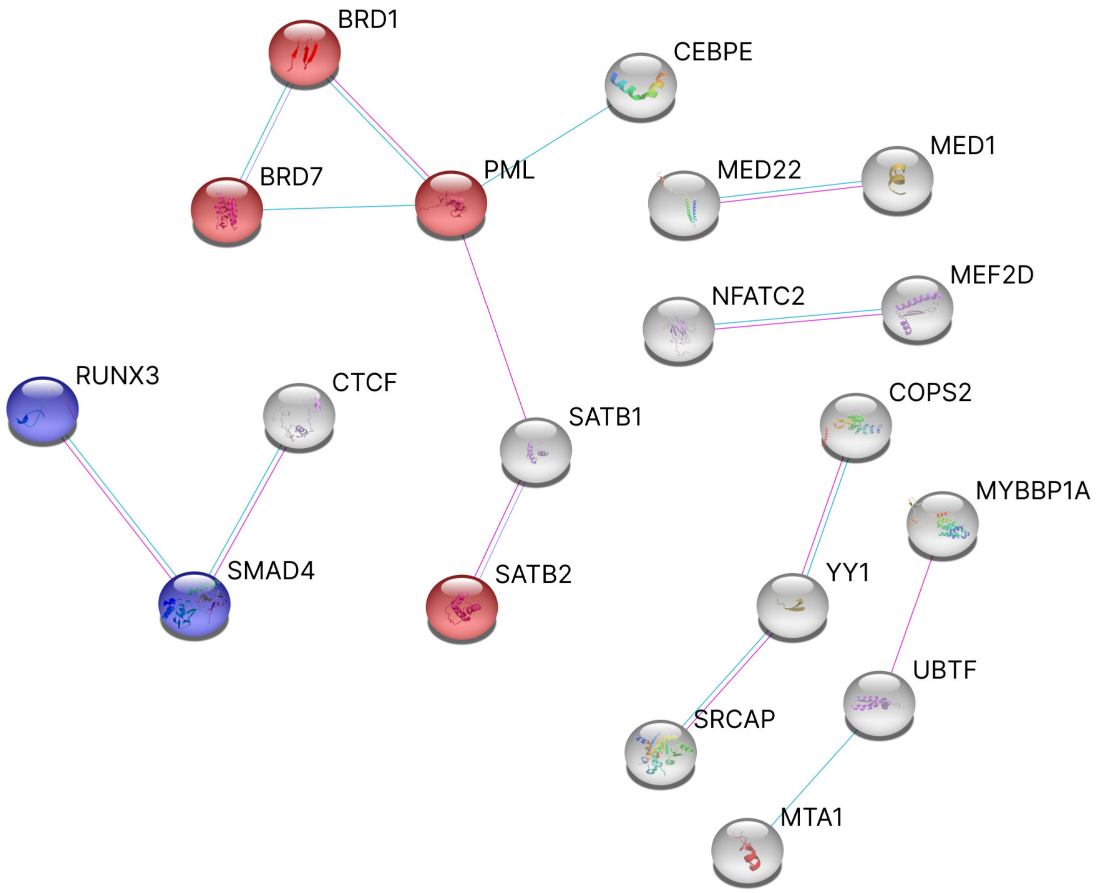

Figure S4. The results of the interaction analysis of 42 downregulated TFs by STRING. Protein dataset was enriched with interactions (PPI enrichment p-value: PPI enrichment p-value: 0.000786, experiments and databases as active interaction sources with the medium confidence score (0.4)) Downregulated TFs were involved in Regulation of TP53 Activity through Acetylation (HSA-6804758) (indicated by color red) and in BCL2L11 (BIM) transcription regulated by RUNX3 (HSA-8952158) (indicated by color blue) according Reactome Pathway.

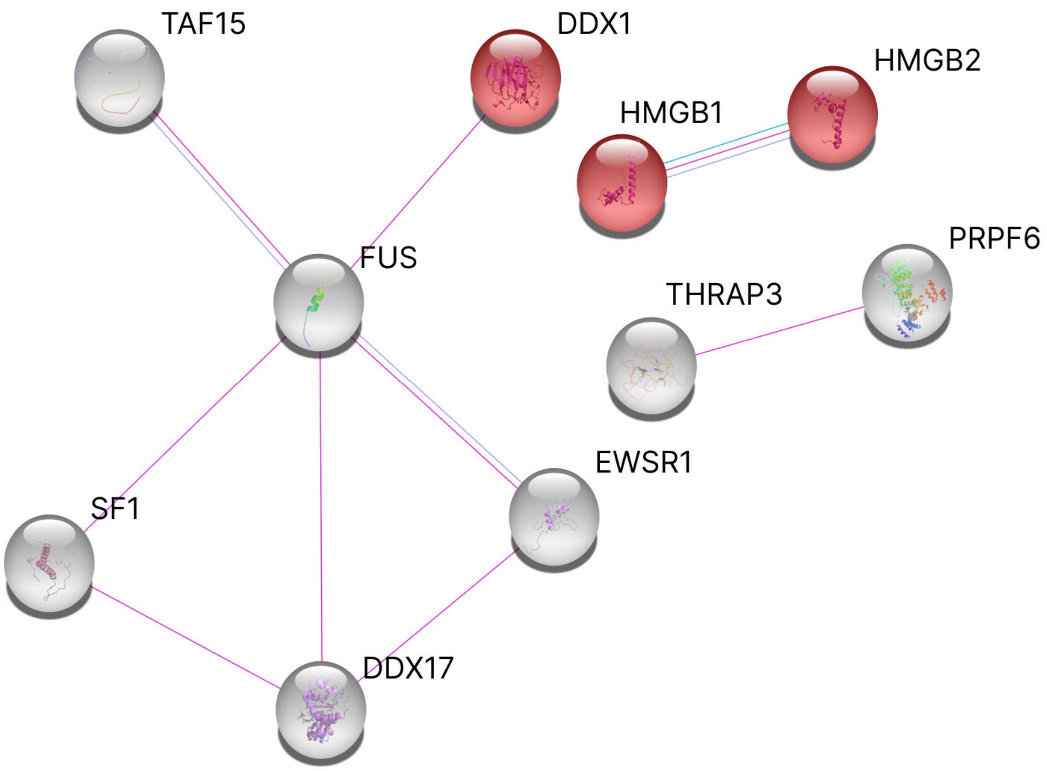

Figure S5. The results of the interaction analysis of 33 upregulated TFs by STRING. Protein dataset was enriched with interactions (PPI enrichment p-value: PPI enrichment p-value: 0.000878, experiments and databases as active interaction sources with the medium confidence score (0.4)). Upregulated TFs were involved in DNA repair (GO:0006281) (indicated by color red).

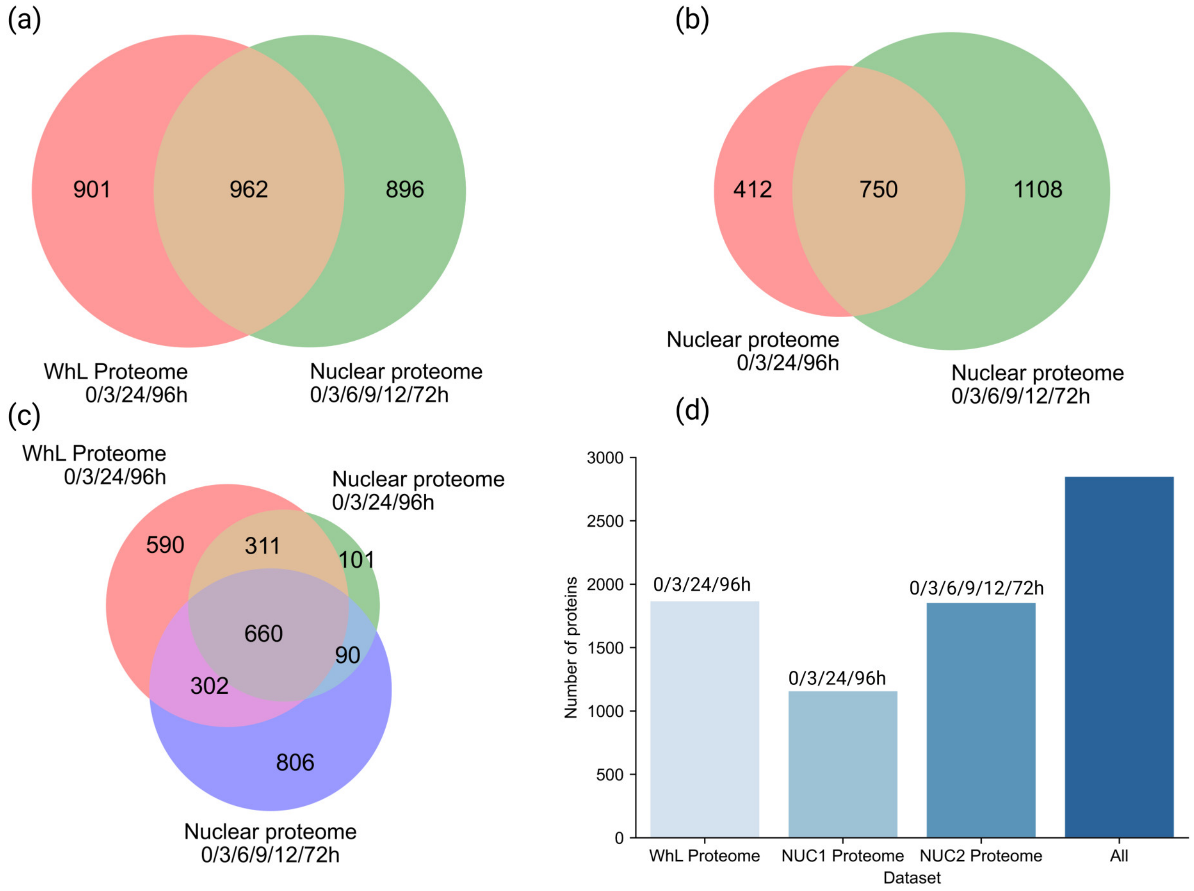

Figure S6. Contribution of mass-spectrometric datasets into coverage of proteomes of HL60 cell line under the ATRA treatment. (a) Venn diagram showing intersection of proteins identified in HL60 cell whole lysate at 0, 3, 24, and 96h after the ATRA treatment (WhL Proteome)[1] and in HL60 nuclear fraction at 0, 3, 6, 9, 12 and 72h after the ATRA treatment (NUC2 Proteome) (current study). (b) Venn diagram showing intersection of proteins identified in HL60 nuclear fraction at 0, 3, 24, and 96h after the ATRA treatment (NUC1 Proteome) [2] and in NUC2 Proteome (current study). NUC1 proteome was obtained in label-free experiment without fractionation in our previous study [2]. NUC2 proteome was obtained in TMT-labeling experiment with alkaline fractionation in current study. (c) Venn diagram showing intersection of proteins (c) and bar chart demonstrating number of proteins identified all three datasets.

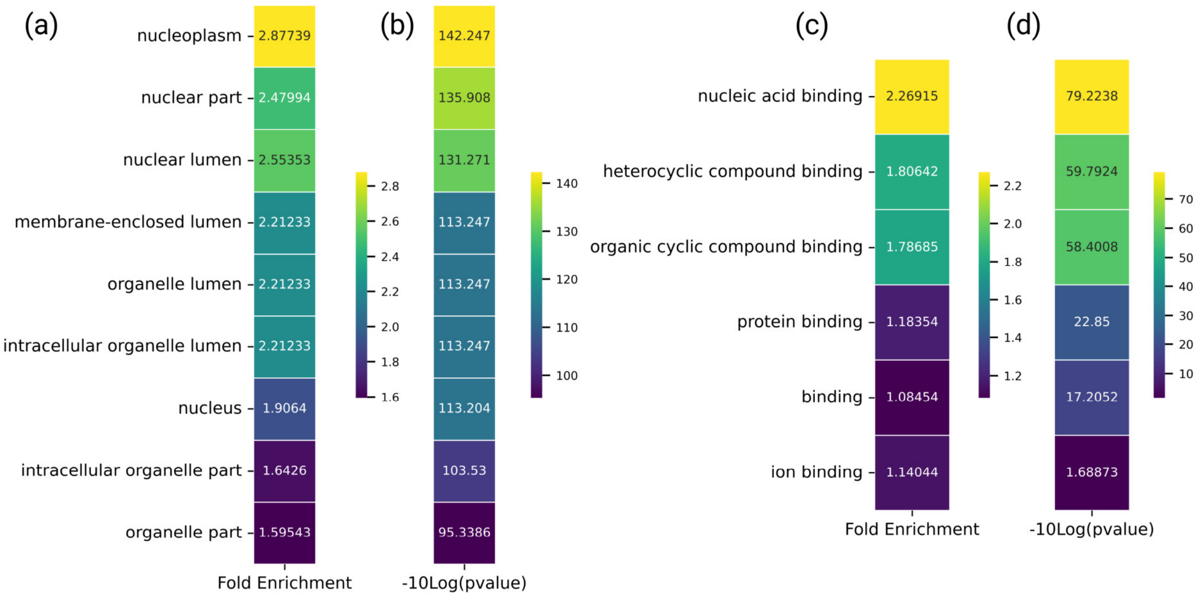

Figure S7. The 1D-heatmap visualization of the functional annotation of 806 nuclear proteins (Figure S6), that were uniquely identified in present study compared to previously investigated HL60 proteomes [1,2]. Cellular Localization (CC) and Molecular Function (MF) were obtained with the DAVID v.6.8. The CC GO and MF GO terms were ranged by enrichment fold (a-c) and by the negative value of the decimal logarithm of p-value Benjamini-Hochberg corrected for multiple testing (b-d), respectively. The CC GO and MF GO terms encompassed at least 300 and 500 proteins, respectively.

20. Novikova, S.; Tikhonova, O.; Kurbatov, L.; Farafonova, T.; Vakhrushev, I.; Lupatov, A.; Yarygin, K.; Zgoda, V. Omics technologies to decipher regulatory networks in granulocytic cell differentiation. *Biomolecules* **2021**, doi:10.3390/biom11060907.

21. Vakhrushev, I. V.; Novikova, S. E.; Tsvetkova, A. V.; Karalkin, P. A.; Pyatnitskii, M. A.; Zgoda, V. G.; Yarygin, K. N. Proteomic Profiling of HL-60 Cells during ATRA-Induced Differentiation. *Bull. Exp. Biol. Med.* **2018**, *165*, doi:10.1007/s10517-018-4210-y.
